# Powder Interlayer Bonding of Nickel-Based Superalloys with Dissimilar Chemistries

**DOI:** 10.3390/ma14082029

**Published:** 2021-04-17

**Authors:** Olivia Stanners, James Russell, Sean John, Helen M. Davies, Silvia Marchisio

**Affiliations:** 1 Institute of Structural Materials, Bay Campus, Swansea University, Swansea SA1 8EN, UK; s.e.john@swansea.ac.uk (S.J.); h.m.davies@swansea.ac.uk (H.M.D.); 2 Advanced Imaging of Materials Facility, Bay Campus, College of Engineering, Swansea University, Swansea SA1 8EN, UK; j.e.russell@swansea.ac.uk; 3 Rolls-Royce plc, P.O. Box 31, Derby DE24 8BJ, UK; Silvia.Marchisio2@Rolls-Royce.com

**Keywords:** powder, interlayer, bonding, joining, nickel-based superalloys

## Abstract

Novel joining methods are crucial for the aerospace industry to repair components damaged in the high stress, high cycle environment of the jet turbine engine. Powder interlayer bonding (PIB) is a novel joining technique that is being explored for use in the aerospace industry. PIB involves the use of a powder interlayer between two faying surfaces alongside a localised temperature gradient and compressive force to produce one joined workpiece. The use of a localised temperature gradient not only reduces the heat affected zone (HAZ) but also reduces the energy requirements for the process as only a small area of the component needs to be elevated in temperature. Nickel-based superalloys are commonly used in the gas turbine engine due to their superior mechanical properties that are maintained even under the most elevated temperatures experienced in the jet turbine engine. It is therefore essential these alloys can be easily repaired. Conventional joining methods such as friction welding have proved difficult for new generation nickel-based superalloys; therefore, there is much interest in PIB as an alternative repair technology. This study shows the potential of PIB to join dissimilar nickel-based superalloys: bonds with very little porosity were observed after only a short processing time.

## 1. Introduction

Nickel-based superalloys are a fundamental material for gas turbine engine manufacture due to their high temperature strength and oxidation resistance that can be maintained at temperatures of up to 1200 °C [1]. This makes the alloys suitable for applications within the harshest of environments such as those experienced in the compressor and turbines within a jet engine [2].

Friction welding is a traditional method that is often used for joining and repair within the aerospace industry. It is considered a solid-state joining method that produces high integrity welds by frictional heat being generated through the movement of one component relative to the one it is being joined to under a force [3,4]. However, new generation nickel-based superalloys have been specifically designed with increased volume fraction of the second phase strengthening precipitate gamma prime (γ′) (>40%) to meet the high temperature requirements of the engine [5]. This increased γ′ increases the strength of the alloy but also hinders weldability by making the material prone to microcracking during weld solidification [6].

Diffusion bonding is a solid-state bonding process that involves the joining of two components without the use of any filler material and pressure and an elevated temperature are used to produce the joint [7,8,9]. The elevated temperatures the materials are subjected to are below the melting point of the materials and this ensures that the process remains solid-state. The joined workpiece is a result of the diffusion of the interface atoms between the bonded materials [10].

Diffusion brazing involves the use of a filler metal between two faying surfaces that are to be joined. The liquidus temperature of the filler metal is lower than that of the faying surfaces; therefore, when the surfaces are heated they remain solid state whereas the filler metal will be in a liquidus state. Similar to diffusion bonding, diffusion brazing also relies on the diffusion of atoms as the liquidus filler metal interacts with the parent materials and diffuses to create the joint [11,12].

Powder interlayer bonding (PIB) is a new technology of interest for jet engine repair due to its ability to produce high-integrity joints at low cost. Indeed, its novelty provides the opportunity to explore the repair of components that would otherwise not be salvaged [13]. PIB has aspects similar to both diffusion bonding and diffusion brazing. The process involves the use of a powder interlayer between two faying surfaces that helps reduce the number of voids among the microscopic asperities between the surfaces to be joined and the reduction in these voids increases the contact area between the surfaces hence, increasing the diffusional area for a bond to be formed. Unlike diffusion brazing, this interlayer does not change in state and remains solid throughout, alongside the parent metals being joined. This means that PIB is a solid-state joining process that relies on the diffusion of atoms between the interlayer powder particles and the faying surfaces to produce a high-integrity joint. The surfaces are subjected to a compressive force and localised heating via induction in an inert atmosphere to promote diffusion of atoms and produce one joined workpiece. PIB has successfully been used to join the titanium alloys Ti-6Al-2Sn-4Zr-6Mo and Ti-6Al-4V [13,14] and a new generation nickel-based superalloy (NGSA) [15].

The joining of dissimilar metals is an important consideration for jet engine design and manufacture; however, it has proved challenging through traditional welding techniques due to the requirement to maintain mechanical performance and the defects that can be present in joined parts often negatively impacting on the mechanical performance compared to the base material mechanical properties [16,17]. There are advantages of using homogenous dissimilar metal components as it provides the possibility to produce workpieces that exploit the advantages of different alloys at their most favourable position within the component [18]. Therefore, new technologies for repairing jet engines where dissimilar metals are required to be joined are crucial for the aerospace industry as many repair methods for these parts are inflexible and do not maintain the mechanical properties of the repaired components, therefore can result in components being scrapped rather than repaired and reused [11,19]. 

## 2. Materials and Methods 

**Figure 1:**
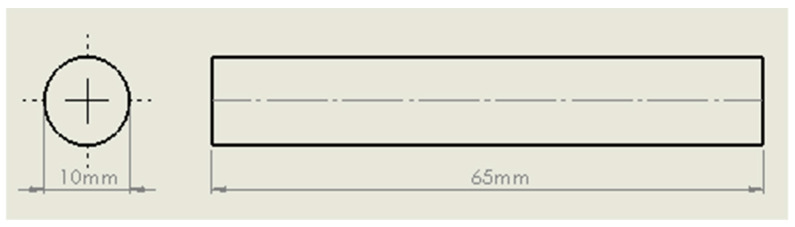
Specimen dimensions.

This study investigated PIB as a joining method of multiple nickel-based superalloys; Inconel 718, RR1000 and a new generation superalloy (NGSA) that is currently being developed by Rolls-Royce Plc (Derby, UK). All the specimens bonded were of uniform geometry with a 10 mm diameter faying surface and 65 mm length as shown in Figure 1.

Powder interlayers were prepared by combining superalloy powder with glycerol that was dipped onto the surface of the specimen. A thin layer of powder then fixed to the specimen surface and any excess removed to leave a powder interlayer of <10 µm thickness. The glycerol then evaporated before the PIB process began. 

**Figure 2:**
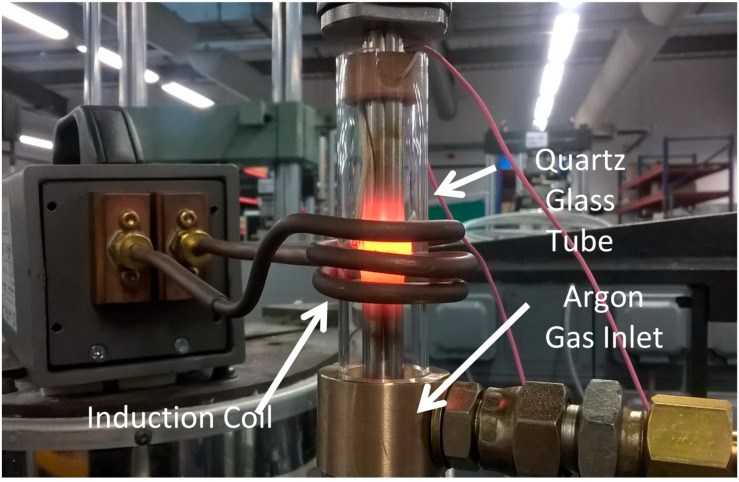
PIB rig setup.

The specimens were secured in an electric screw rig via collets and their faying surfaces aligned until touching with a low force <0.5 kN. A water-cooled induction coil was then positioned around the specimens in the powder interlayer region to create localised heating. The induction coil then heated the localised area to temperatures of 970–1050 °C. The bonding temperature was measured using N-type thermocouples that had been spot-welded onto the top specimens within 1 mm of the faying surfaces. The thermocouples were connected to a PID control system with accuracy within 1 °C, that allowed the temperature to accurately be controlled throughout the PIB process. Once the interlayer region was heated to the desired temperature, a compressive force of 5–6 kN was applied via the electric screw rig. The desired temperatures were determined from results of previous studies involving nickel-based superalloys [7]. All PIB bonds were performed in an argon environment to prevent oxidation during the bonding process. The use of an argon inlet collet and glass tube with a metal collet ensured a constant flux of argon to the specimen. The PIB rig set up is shown in Figure 2 and the parameters for each bond investigated in this study are shown in Table 1.

The specimens were held under load and at temperature for 30 min. The parameters for each bond completed are shown in Table 1. 

Once the specimens were successfully bonded into one workpiece they were left in the rig and allowed to air cool before being removed. The percentage deformation is displayed in Table 2 and was measured at the interlayer region by calculating the change in diameter and length using:

(1) $$Deformation\;\left(\%\right)=\left(\frac{Change\;in\;diameter/length}{Original\;diameter/length}\right)\times 100$$

Post bonding, joints were sectioned around the interlayer region and mounted in conductive Bakelite ready for microstructural analysis. The specimens were subjected to standard grinding and polishing sequences. 

Scanning electron microscope (SEM) images of the bondline region were taken using back-scattered electron (BSE) imaging on a Hitachi SU3500 SEM (Hitachi, Tokyo, Japan). 

SEM images of the bondline at the same magnifications were used to obtain porosity measurements. Porosity measurements were acquired through ImageJ software. The ‘threshold’ function was selected and set for each bondline. The software then calculated both the number and area of pores present. 

All field emission gun scanning electron microscopy (FEG-SEM) images were captured once the specimens had been electro-etched using a 10% phosphoric acid solution. The images were taken using a JEOL 7800 FEG-SEM (JEOL, Tokyo, Japan).

**Figure 3:**
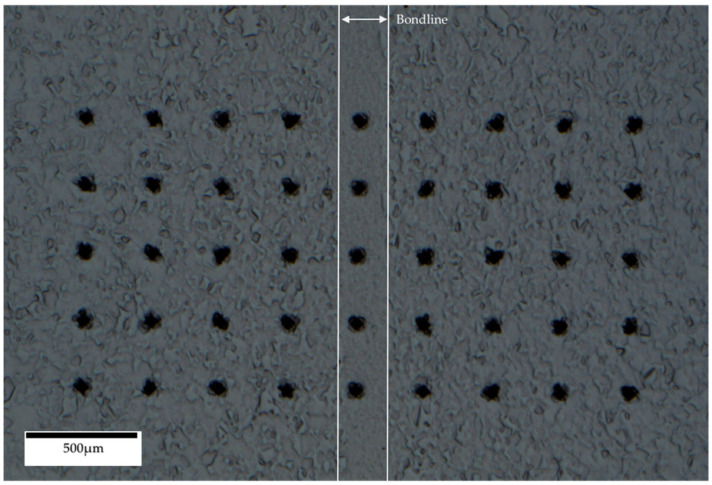
9 × 5 Vickers Hardness indent grid.

Vickers microhardness data was obtained using a Struers Duramin-40 A3 machine (Struers, Copenhagen, Denmark). A 9 × 5 grid with 0.3 mm spacing was constructed allowing 45 indents to be made and measured. Microhardness testing standards require indent spacing of at least 3 times the indent length in order to avoid false results due to work hardening effects. The 5th indent on each row coincided with the bondline of each specimen. The hardness measurements used a 1 kgf and a 10 s dwell time. The grid constructed for the hardness analysis is shown in Figure 3.

## 3. Results and Discussion

### 3.1. Base Materials

**Figure 4:**
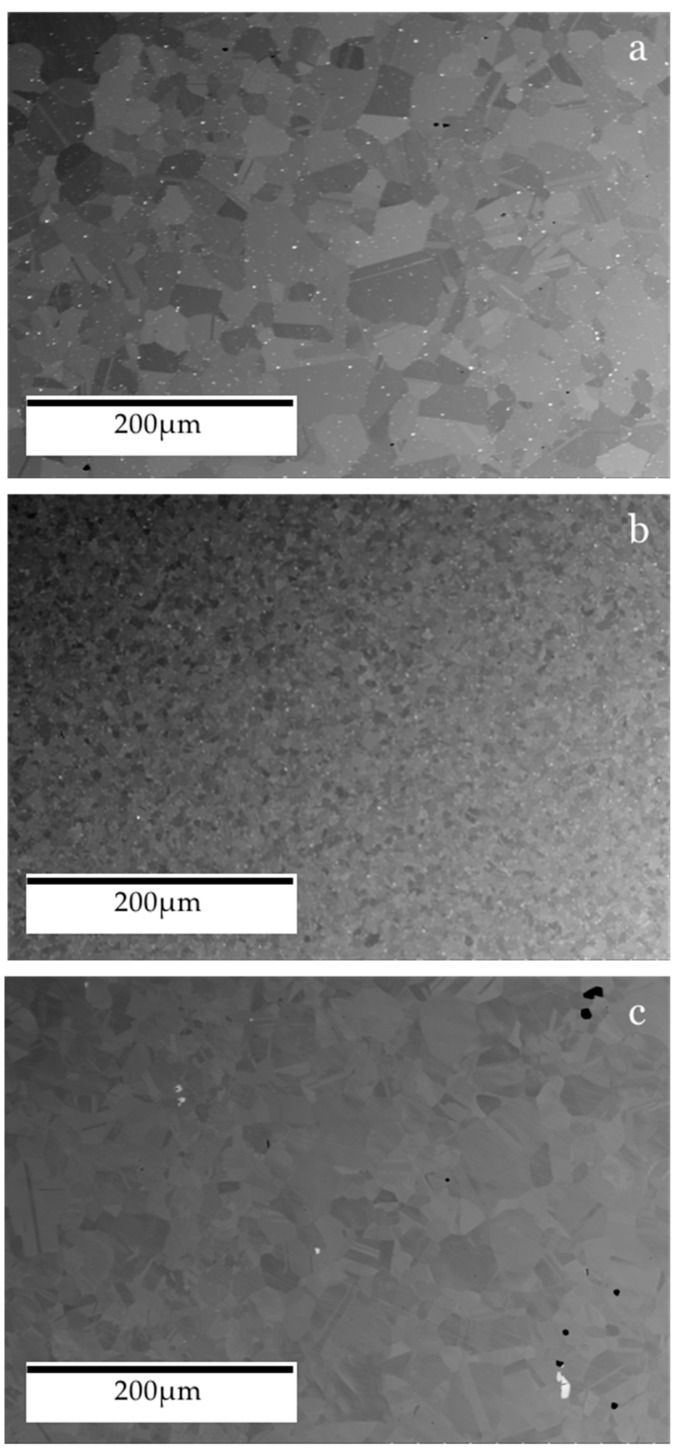
SEM BSE of base materials: (**a**) new generation nickel-based superalloy, (**b**) RR1000, (**c**) Inconel 718.

The as-received pre-bonded microstructure of the nickel-based superalloys investigated is shown in Figure 4. As these alloys are gas turbine disk materials, they have been designed to withstand the most elevated temperatures in the jet engine while retaining a high degree of strength. The main strengthening mechanism associated with nickel-based superalloys is a precipitation strengthening, in the form of a second phase [20]. For RR1000 and the NGSA examined, this strengthening precipitate is the γ′ phase while Inconel 718 is strengthened with the gamma double prime (γ″) phase. In all the alloys investigated, this strengthening precipitate phase exists within a face-centred cubic (FCC) matrix phase of gamma (γ) [21,22]. The γ′ phase is FCC whereas the γ″ phase is body-centred tetragonal (BCT); however, both phases act to strengthen the alloy by impeding the movement of dislocations throughout the body of the material. The γ″ present in Inconel 718 conveys additional strength compared to γ′ in RR1000 and the new generation nickel-based superalloy. The additional strength of the metastable γ″ is compromised at the highest of temperatures (> 650 °C) as it decomposes into the δ phase that is thermodynamically stable [23].

**Figure 5:**
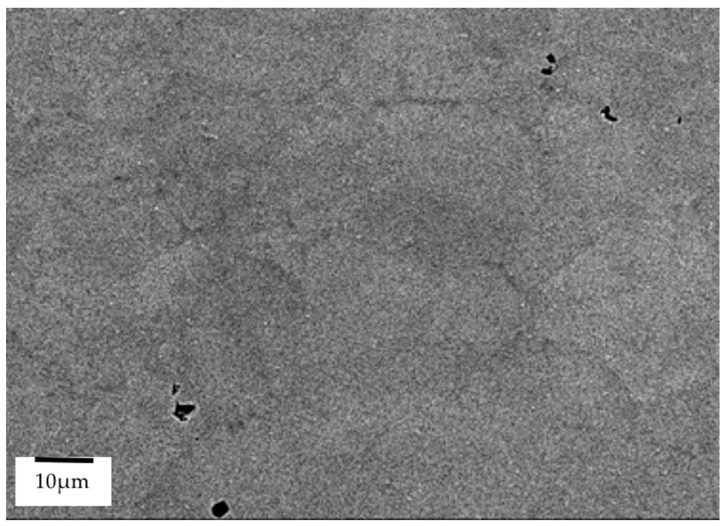
FEG-SEM of pores in as-received new generation nickel-based superalloy.

From Figure 4 it is possible to observe the variation in grain size between the different alloys. The RR1000 has a much smaller grain size (4–11 µm [24]) than Inconel 718 (58 µm [25]) and the NGSA (23 µm [7]). Interestingly, a large variation of grain size within the microstructure of the NGSA is present with grains as small as 5.5 µm and as large as 76 µm being found during analysis. A number of pores are also present within the pre-bonded as-received microstructure shown in Figure 5, these are a result of the current powder metallurgy manufacturing process of the alloy.

### 3.2. Grain Size

**Figure 6:**
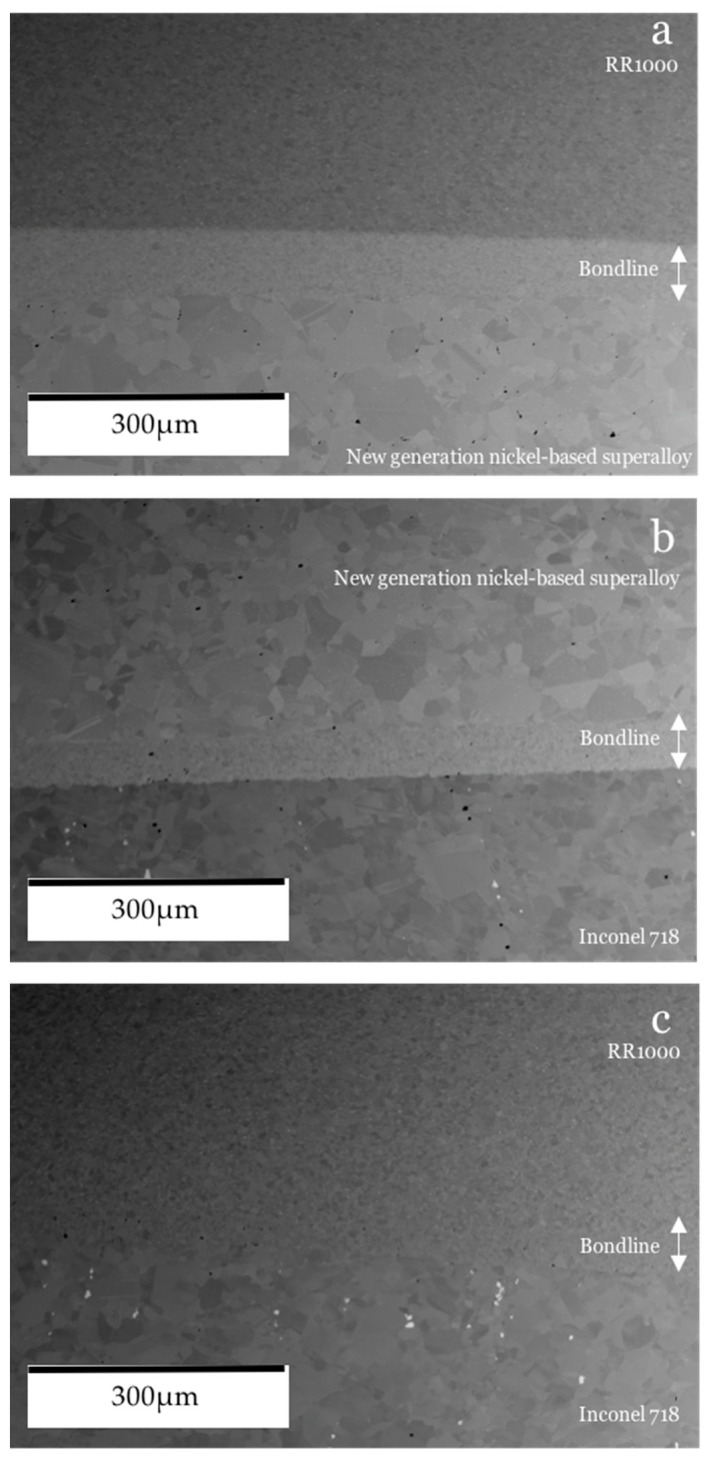
SEM BSE of bonds: (**a**) bond 1, (**b**) bond 3, (**c**) bond 3.

Figure 6 shows the appearance of the bondline compared to its surrounding base materials in each of the dissimilar bonds created. Figure 6a shows the results of bond 1 which involved the γ′ strengthened alloys RR1000 and the NGSA and an NGSA powder interlayer. The bondline has a microstructure consisting of much finer grains than the NGSA base material which has considerably coarser grains and the presence of pores can be observed within the bulk. The RR1000 base material, however, appears pore-free and to have fine grains, much like the bondline. The bondline appears to be relatively pore-free. Figure 6b shows bond 2 which comprised of the NGSA joined to Inconel 718 via a NGSA powder interlayer. The base materials of this bond both have a coarser grain size than RR1000 and both appear to have pores. The resultant bondline for these materials is much finer in grain size than the surrounding base materials and pores within the bondline can be observed. Figure 6c shows the bondline of bond 3 where RR1000 was joined to Inconel 718 with an RR1000 powder interlayer. The bondline is very similar in appearance to the surrounding RR1000 base material, but pores within the bonded area are present whereas the RR1000 parent material does not appear to have any pores. It is evident that during bonds 1 and 2 the NGSA has maintained its much coarser grain size which was observed in the base material before bonding. The fine grain bondline compared to the surrounding base material has also been observed in other PIB trials involving the NGSA [7]. 

### 3.3. γ′/γ″ Morphology

**Figure 7:**
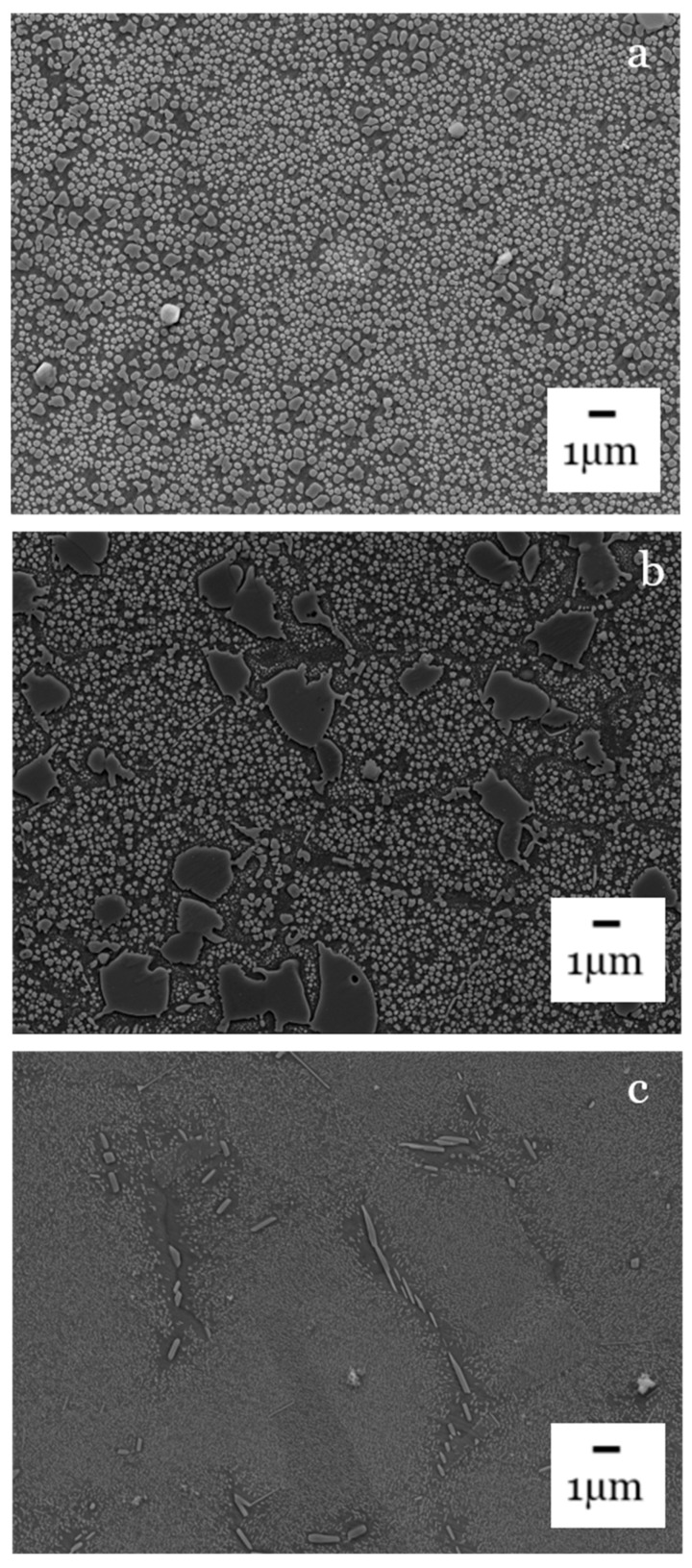
FEG-SEM of base materials: (**a**) new generation nickel-based superalloy, (**b**) RR1000, (**c**) Inconel 718.

**Figure 8:**
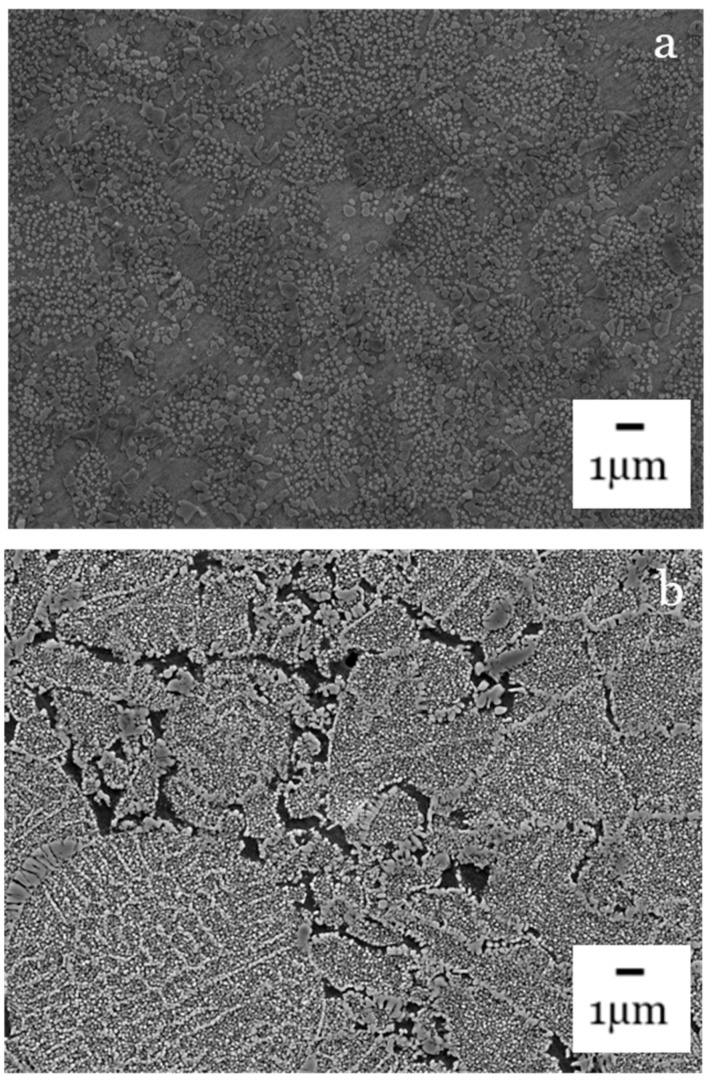

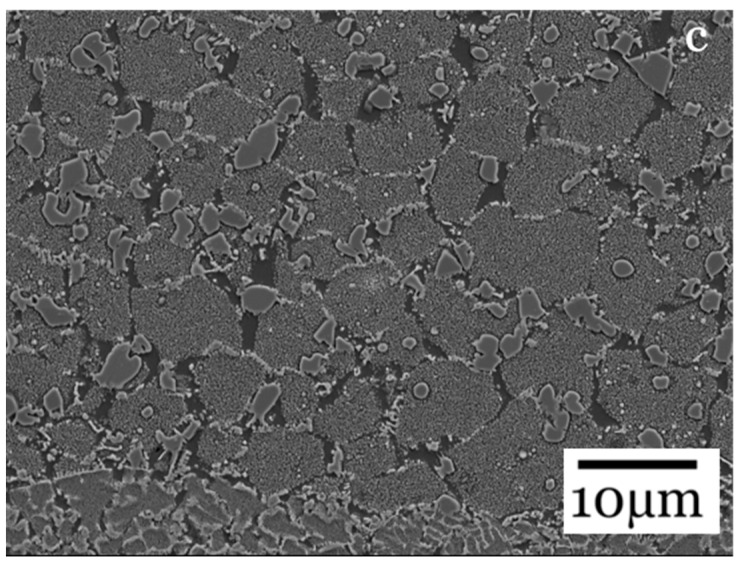
FEG-SEM of bondlines: (**a**) bond 1, (**b**) bond 2, (**c**) bond 3.

Figure 7 and Figure 8 show the morphology and distribution of strengthening precipitates and it can be seen how these vary throughout the interlayer region and differ between the dissimilar base materials. In Figure 7a large volume of secondary γ′ can be observed in the NGSA and this will act to strengthen the alloy by impeding the movement of dislocations. RR1000 is shown to have both primary and secondary γ′ precipitates present and the primary are much larger in size than the accompanying secondary precipitates. Inconel 718 has a much sparser distribution of its strengthening precipitates compared to the other alloys however these precipitates are γ″ rather than γ′.

Figure 8 shows the bondlines. In Bond 1 where both alloys are γ′ strengthened, the bondline appears to have a consistent distribution of γ′ precipitates smaller in size than in the base materials. This, however, is the region that has been subjected to the highest temperatures and as a result there are areas that appear to be free of γ′. This is likely due to the precipitate dissolving into the background γ matrix at the elevated bonding temperatures. For bond 2, the outline of the individual powder particles that have been used to create the interlayer can be observed. There are also porous areas between these powder particles which are undesirable. It appears the powder has not fully collapsed [6] or fully diffused into the bondline. This indicates both a higher bonding force and temperature would be beneficial to create a pore free bondline as would be required for joining and repair purposes. The bond 3 bondline has γ′ strengthening precipitates, however there are areas that appear to be free from any strengthening precipitates which is not desirable when mechanical performance is considered; nonetheless, the bondline appears to be relatively pore free which is preferable for a joint in material.

### 3.4. Bond Microhardness

**Figure 9:**
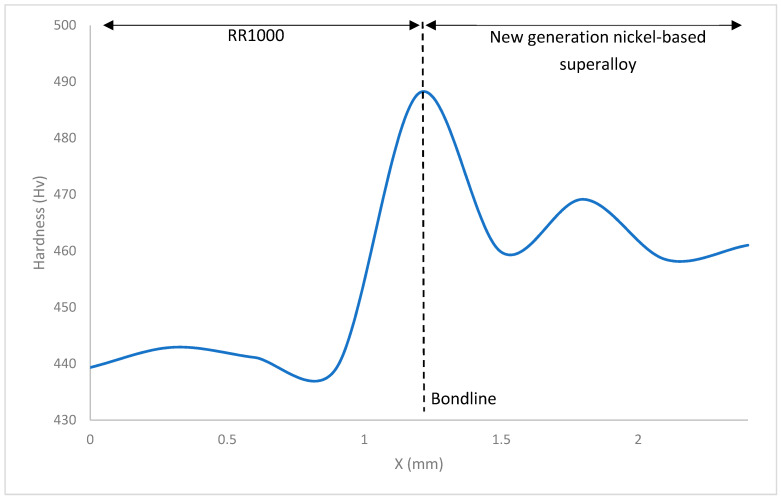
Graph detailing Vickers Hardness variation in bond 1.

**Figure 10:**
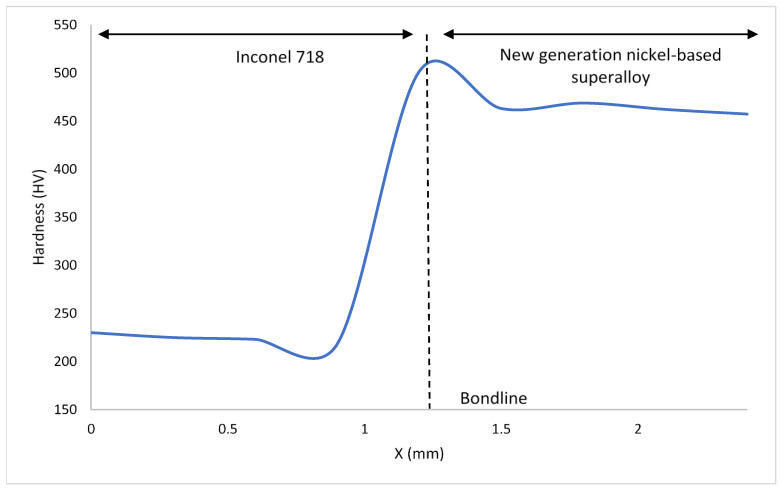
Graph detailing Vickers Hardness variation in bond 2.

**Figure 11:**
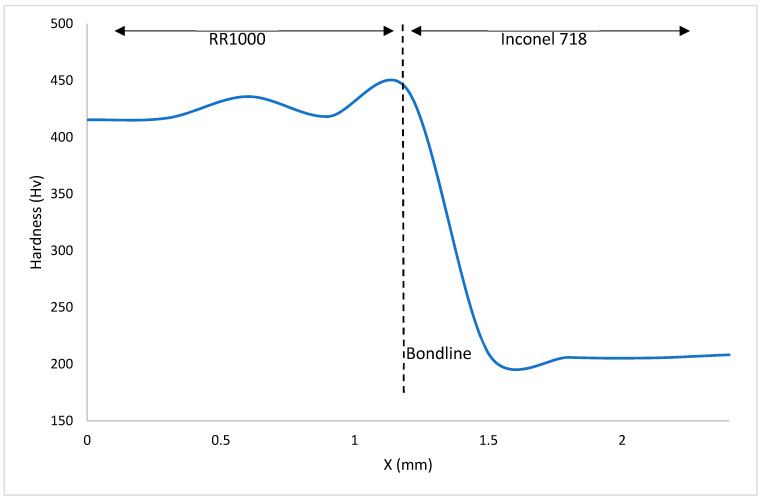
Graph detailing Vickers Hardness variation in bond 3.

Figure 9, Figure 10 and Figure 11 show the hardness values observed throughout the HAZ (including the bondline) and into the base materials for the bonds investigated. The bondline is represented at X = 1.2 mm. 

For all the bonds, the highest values of hardness were observed at the bondline with hardness values as high as 501 HV seen in Figure 9. These areas where the highest hardness values have been obtained correlate to where the finest grains have been observed (Figure 6). These finer grained microstructures will contain more grain boundaries and therefore have a larger total grain boundary area compared to the coarse microstructures. Since grain boundaries act to impede dislocation movement, the microstructures with higher total grain boundary areas will therefore hinder more dislocation movement and therefore result in higher hardness values for these microstructures. Hardness measures a materials resistance to plastic deformation and therefore correlates to the materials yield stress. The relationship between yield strength and average grain size is shown in the Hall–Petch relationship:

(2) $$\sigma_{y}=\sigma_{0}+k_{y}d^{-\frac{1}{2}}$$

where $\sigma_{y}$ is the materials yield stress, $\sigma_{0}$ is the lattices resistance to dislocation movement, $k_{y}$ the materials strengthening coefficient that is specific to each individual material, d is the average grain diameter in the microstructure [26,27].

For all the bonds there is a decrease in hardness as the distance from the bondline and HAZ is increased. Similar trends to this have been observed in inertia friction welding (IFW) of nickel-based superalloys [28]. Dissolved γ′ in areas close to the bondline re-precipitates during cooling and the resultant γ′ fine precipitates act to strengthen the alloy in these areas, resulting in the increase in hardness observed in the HAZ. As the bonds were allowed to air cool, there was time for precipitation to occur, however as they were not subjected to post-bond heat treatments, the time for any particle growth is limited. It is widely reported the benefits of fine sized γ′ precipitates in strengthening nickel-based superalloys and as these alloys are known to gain their mechanical properties primarily through strengthening precipitates, it has been observed that hardness values higher than parent materials can be present after bonding or welding processes where a fine size distribution of γ′ precipitates has formed [29]. 

When the hardness of the material surrounding the bondline is analysed, the material that displays the highest values is the NGSA with hardness as high as 469HV. This is not surprising as it is known that this alloy has been designed with a large volume of γ′ strengthening precipitates. From Figure 4, the presence of twin crystal boundaries can be observed and these will also act to impede dislocation movement and increase the hardness of the material. 

Inconel 718 is shown to have the lowest hardness values. Hardness values as low as 205 HV are a characteristic of a decreased volume of strengthening precipitates compared to the other materials analysed. As Inconel 718 has been bonded at temperatures greater than 650 °C, its strengthening γ″ phase will have begun to transform into the more stable δ phase during the bonding process and hence a smaller volume of strengthening γ″ precipitates will be present within the microstructure [23].

### 3.5. Porosity

From Table 3 and Table 4, it can be seen that RR1000 was the least porous material both as-received prior to bonding and as a surrounding base material when bonded. As received, the material had 0.006% area porosity compared to 0.037% and 0.025% for NGSA and Inconel 718, respectively. 

**Figure 12:**
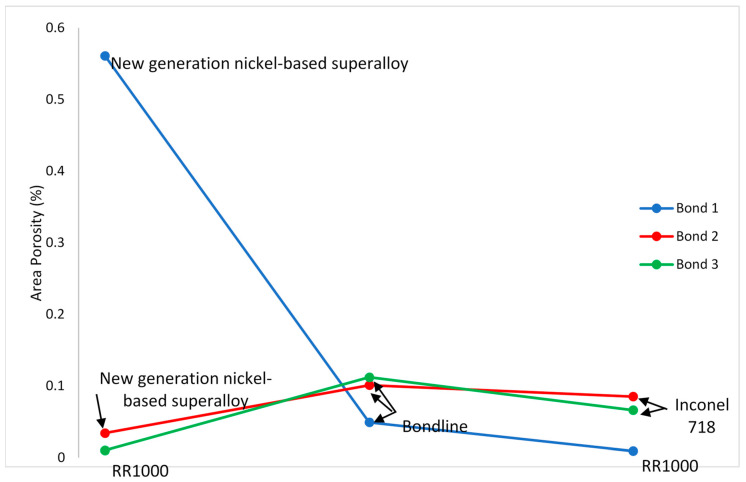
Graph detailing porosity variation throughout bonds.

When the bondline versus surrounding base material results are considered, bond 1 has a lower percentage area porosity than the surrounding base material with 0.049% compared to 0.561% porosity in the NGSA surrounding the bondline. Bonds 2 and 3 both had more porosity in the bondline than the surrounding base material with a percentage area porosity of 0.101% in bond 2 with the surrounding Inconel 718 being only 0.085% porous and bond 3 having 0.112% porous area compared to 0.66% in the Inconel 718 surrounding its bondline as seen in Figure 12.

As a surrounding base material, Inconel 718 was the most porous with up to 0.085% of its area being porous. As received, NGSA has the highest area porosity of 0.037%. 

## 4. Conclusions

The following conclusions were drawn from this programme of research:

- Bondline regions have a much finer microstructure with an increased volume of grains that are smaller in size than those of the base materials bonded.
- The bondline region provides an area of increased hardness and this relates to the Hall–Petch relationship.
- An increased volume of finer precipitates at bondlines and the nearby HAZ contributes to increased hardness in these regions and this is a trend also observed in inertia friction welding of nickel-based superalloys.
- γ′ strengthened alloys proved to be harder than γ″ strengthened alloys after being subjected to elevated bonding temperatures as the γ″ strengthening precipitates decompose into the stable δ phase.
- There is no clear trend between porosity in the bondline and surrounding base material for PIB of dissimilar materials as bondlines with reduced porosity compared to their surrounding material and bondlines with increasing porosity compared to their surrounding materials were both observed in this study.
- Further work is required to understand fully the mechanical property effects of PIB for dissimilar metals.

## Figures and Tables

**Table 1:** Dissimilar bonding parameters.

Bond	Specimen 1	Specimen 2	Time (min)	Temperature (°C)	Force (kN)	Interlayer
**1**	New generation nickel-based superalloy	RR1000	30	1050	5	New generation nickel-based superalloy
**2**	New generation nickel-based superalloy	Inconel 718	30	970	6	New generation nickel-based superalloy
**3**	RR1000	Inconel 718	30	970	6	RR1000

**Table 2:** Length and diameter deformation of bonds.

Bond	Length Deformation	Diameter Deformation
**1**	−2.659%	46.3%
**2**	−0.013%	5.5%
**3**	−1.415	11.5%

**Table 3:** Base material porosity.

As-Received Material	% Area Porous
New generation nickel-based superalloy	0.037
RR1000	0.006
Inconel 718	0.025

**Table 4:** Bond porosity.

Bond	Material	% Area Porous
**1**	New generation nickel-based superalloy	0.561
	Bondline	0.049
	RR1000	0.009
**2**	New generation nickel-based superalloy	0.034
	Bondline	0.101
	Inconel 718	0.085
**3**	RR1000	0.010
	Bondline	0.112
	Inconel 718	0.066

## Data Availability

Data available on request due to restrictions. The data presented in this study are available on request from the corresponding author. The data are not publicly available due to commercial sensitivity.
